# Supplementary material for: Seminoma and Embryonal Carcinoma Footprints Identified by Analysis of Integrated Genome-Wide Epigenetic and Expression Profiles of Germ Cell Cancer Cell Lines
Source: PLoS One. 2014 Jun 2;9(6):e98330. doi: 10.1371/journal.pone.0098330 (PMC4041891; doi:10.1371/journal.pone.0098330)
Supplement: File S1 — ZIP file containing DMRforPairs output for significant regions. Please start from the html files. (ZIP) [file pone.0098330.s008.zip › figures/30236.pdf]

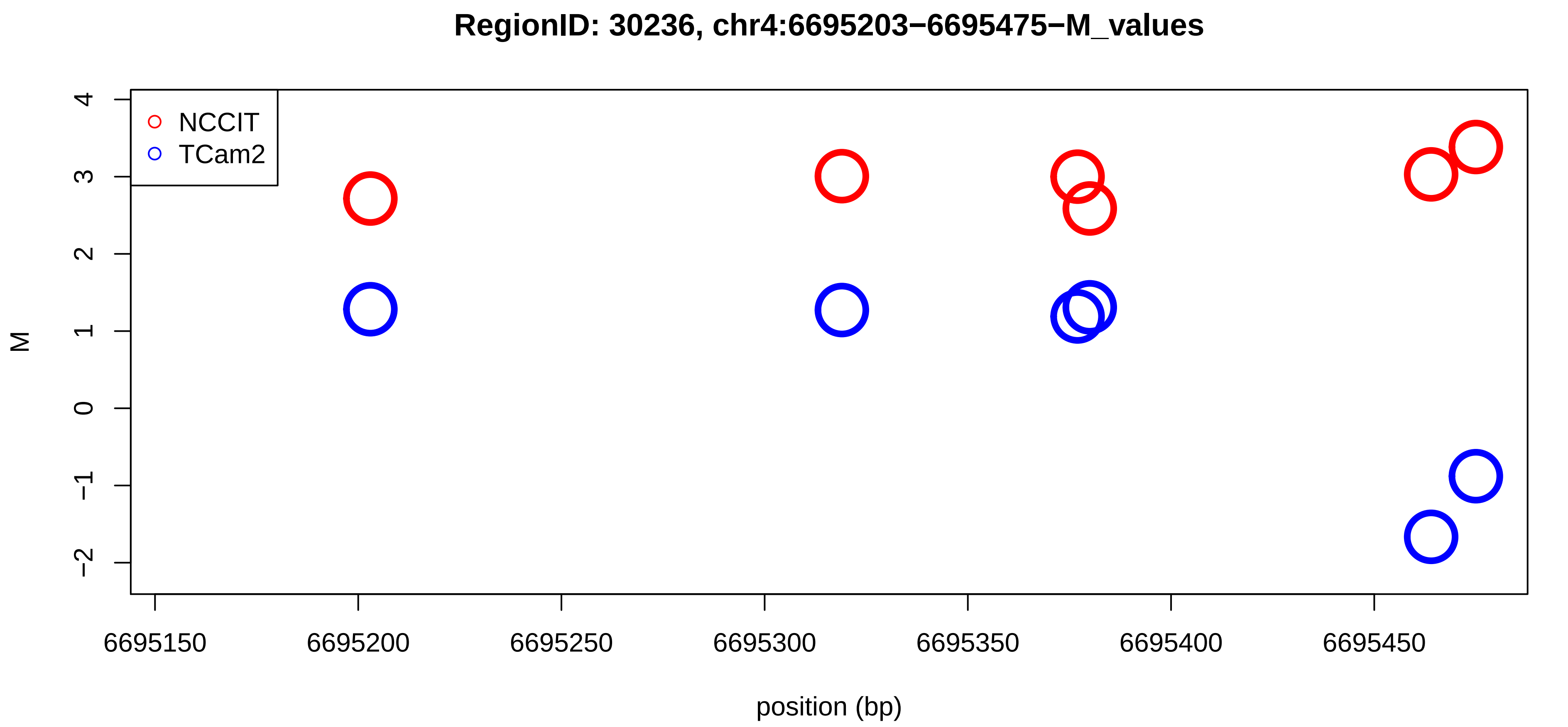
RegionID: 30236, chr4:6695203−6695475−M_values
NCCIT
TCam2
M
−2
−1
0
1
2
3
4
6695150
6695200
6695250
6695300
6695350
6695400
6695450
position (bp)

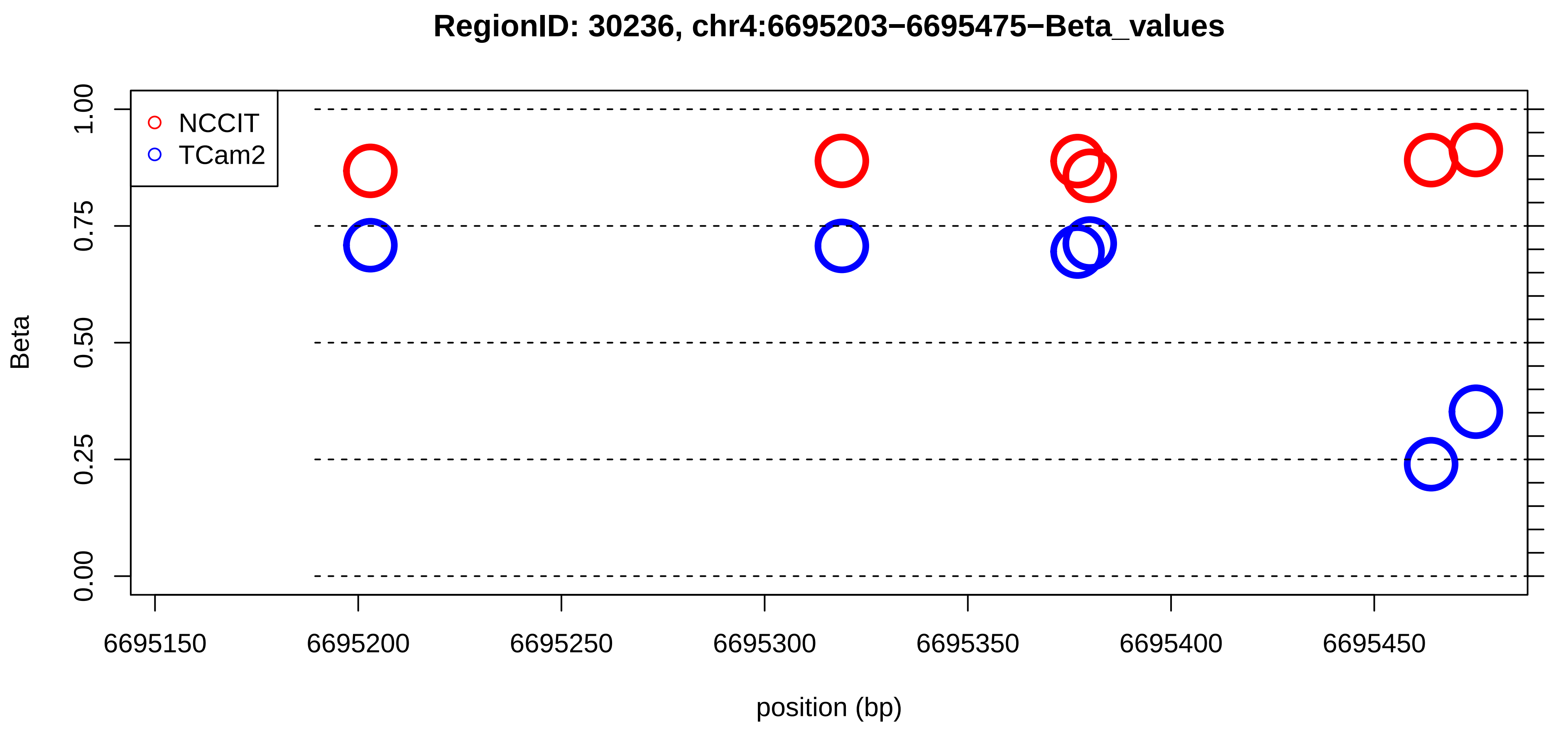
RegionID: 30236, chr4:6695203−6695475−Beta_values
NCCIT
TCam2
Beta
0.00
0.25
0.50
0.75
1.00
6695150
6695200
6695250
6695300
6695350
6695400
6695450
position (bp)
